# Reply to: “Bio-optimized *Curcuma longa* extract is efficient on knee osteoarthritis pain: a double-blind multicenter randomized placebo controlled three-arm study”

**DOI:** 10.1186/s13075-020-2107-4

**Published:** 2020-02-11

**Authors:** Nicola Veronese

**Affiliations:** National Research Council, Neuroscience Institute, Aging Branch, Via Giustiniani, 2, 35128 Padova, Italy

Dear Editor,

I read with interest the work entitled “Bio-optimized *Curcuma longa* extract is efficient on knee osteoarthritis pain: a double-blind multicenter randomized placebo controlled three-arm study” [1].

However, I believe that this work has some important shortcomings. First, this work was registered retrospectively. The study started in 2014, but was only recorded in September 2016, which is more than inappropriate. Second, the co-primary endpoints are arbitrary, and not recommended by any regulatory agency or scientific society [2, 3]. Third, the calculation of the sample size should be checked. The authors calculated the numerosity assuming that the null hypothesis could be true, since the standard deviation of the co-primary endpoints is larger than the mean difference expected between the treated and the placebo groups [4]. A fourth relevant point is that co-primary endpoints fail to show any significant difference, at any time point or at the end of the study, in the intention to treat analysis. This is important also because the last assessment is after 6 months after baseline evaluation, while the authors discussed only 3 months evaluation. However, the 6 months evaluation (as reported in the protocol) contains the same tests made at the baseline and at 1 and 3 months. They should therefore clarify the role of the 6 months tests. Fifth, only post hoc analyses reported a difference compared to placebo group, which is methodologically disputable in a study that fails to meet the primary endpoints. I would like to remember that post hoc analyses are not pre-planned and this approach usually introduces a multiple testing problem. A correction for multiple testing is not mentioned, but is of great importance for the correct interpretation of the results. Finally, the authors claimed that BCL treatment is safe. However, the authors reported a significant increase in gastrointestinal adverse events (especially diarrhea) in the high dose of BCL. It should be noted that 18.5% left the trial for the presence of adverse events in the high BCL group and that this is higher than expected as lost of follow-up (15%). Therefore, we cannot affirm that BCL is a safe treatment of knee OA.

In conclusion, due to the important methodological shortcomings that I reported, I believe that these findings should be taken cautiously not allowing to believe that BCL can be used for the treatment of knee OA and that future studies with a more accurate methodology should be performed.

## Data Availability

Not applicable
